# From the Cochrane Library: Interventions for Cutaneous Molluscum Contagiosum

**DOI:** 10.2196/41514

**Published:** 2023-04-25

**Authors:** Ani Oganesyan, Torunn E Sivesind, Robert Dellavalle

**Affiliations:** 1 University of Colorado School of Medicine Aurora, CO United States; 2 Department of Dermatology University of Colorado Anschutz Medical Campus Aurora, CO United States; 3 Department of Dermatology Rocky Mountain Regional Veterans Affairs Medical Center Eastern Colorado Health Care System Aurora, CO United States

**Keywords:** Cochrane, systematic review, randomized controlled trial, RCT, molluscum contagiosum, dermatology, intervention, skin, dermis, derma, epidermis, dermatologist, poxvirus, skin infection, molluscum, cutaneous, skin lesion

Molluscum contagiosum (MC) is a cutaneous and mucosal condition that primarily affects children and immunocompromised adults. It presents with skin-colored, dome-shaped papules on the skin and may be associated with pain, pruritus, erythema, and, rarely, bacterial superinfection. Although spontaneous resolution generally occurs, treatment may be indicated for cosmetic purposes or to prevent spread.

A 2017 Cochrane systematic review [1] evaluated 22 randomized controlled trials (N=1650 participants, aged 0-36 years) and sought to provide an evidence base supporting specific treatments. Inclusion criteria required participants to have a clinical diagnosis of MC, excluding those with immune deficiency or sexually transmitted MC, as well as assessment of physical ablative methods (curettage, cryotherapy), topical agents (potassium hydroxide, iodine, trichloroacetic acid, salicylic acid, 10% phenol/70% alcohol, tretinoin, oils, cantharidin, podophyllotoxin, imiquimod), and systemic therapy (cimetidine, 35 mg/kg per day; calcarea carbonica, daily for 15 days).

The primary outcome was short‐term clinical cure, defined as the complete disappearance of lesions up to 3 months after the initiation of treatment, as assessed by a physician. The secondary outcomes were clinical cure up to and beyond 6 months, time to cure, recurrences (after 3, 6, and 12 months), adverse effects (pain, blistering, sensitization, scarring, erosion, and pigmentary changes), spread, and disease‐related quality of life.

The treatment comparisons performed in the included studies are summarized in Table 1. Data from this review strongly support awaiting spontaneous resolution of molluscum lesions and demonstrated that 5% imiquimod was no more effective in terms of clinical cure than the placebo (with an identical vehicle). Furthermore, the use of 5% imiquimod was reported to be more harmful regarding application site reactions and no more effective than its vehicle over a 3-month period.

Newer studies have proposed novel treatment options. Notably, a 2020 case study and literature review [2] described the effectiveness of photodynamic therapy (2 sessions, 2 weeks apart using 630-nm red light lasting 9 minutes) in association with incubation with 5’-Aminolevulinic acid in completely resolving giant MC (larger than 1 cm in diameter). An alternate review by Wells et al [3] discussed intralesional immunotherapies in the treatment of MC and highlighted case reports exhibiting resolution rates between 36% and 100% with minimal adverse reactions (erythema, mild edema) with Candida antigen, MMR (measles, mumps, and rubella) vaccine, vitamin D3, and OK- 432 (a penicillin- and heat-treated lyophilized powder of the *Streptococcus pyogenes* A3 substrain). A retrospective cohort study by Chauhan et al [4] assessed 22 patients between the ages of 6 to 50 years treated with 1 to 3 doses of 0.5 ml of intralesional MMR. They found that 18 (81.8%) patients had complete clearance of lesions and 4 (18.18%) patients had a partial response of more than 50% clearance. This benefit was observed in both injected and distant lesions in both studies.

Limitations of these studies include their observational design and lack of a control group. Furthermore, these studies did not equally delineate the time frame in which the participants experienced the lesions, nor whether the participants’ results were affected by other dermatologic diagnoses. There is a need for larger, placebo-controlled, and prospective studies using both intralesional immunotherapy and phototherapy to confirm their efficacy.

**Table 1:** Treatment comparison with respective results and statistics.

Comparison	Measurement	Result	Statistics
5% imiquimod vs cryospray	Physician assessment	Cryotherapy was superior	1 study, N^a^=74; RR^b^ 0.60, 95% CI 0.46-0.78
5% imiquimod vs 10% potassium hydroxide	Physician assessment	Potassium hydroxide was superior	2 studies, N=67; RR 0.65, 95% CI 0.46-0.93
5% imiquimod vs placebo	Physician assessment	Neither intervention was superior	4 studies, N=850; RR 1.33, 95% CI 0.92-1.93
Topical 10% Australian lemon myrtle oil vs olive oil	Physician assessment	10% Australian lemon myrtle oil was superior	1 study, N=31; RR 17.88, 95% CI 1.13-283
10% benzoyl peroxide cream vs 0.05% tretinoin	Physician assessment	10% benzoyl peroxide cream was superior	1 study, N=30; RR 2.20, 95% CI 1.01-4.79
5% sodium nitrite coapplied with 5% salicylic acid vs 5% salicylic acid alone	Physician assessment	5% sodium nitrite coapplied with 5% salicylic acid was superior	1 study, N=30; RR 3.50, 95% CI 1.23-9.92
Iodine plus tea tree oil vs tea tree oil	Physician assessment	Iodine plus tea tree oil was superior	1 study, N=37; RR 0.20, 95% CI 0.07-0.57
Iodine plus tea tree oil vs iodine alone	Physician assessment	Iodine plus tea tree oil was superior	1 study, N=37; RR 0.07, 95% CI 0.01-0.50
Homeopathic calcarea carbonica vs placebo	Physician assessment	Neither intervention was superior	1 study, N=20; RR 5.57, 95% CI 0.93-33.5
2.5% potassium hydroxide solution vs 5% potassium hydroxide solution	Physician assessment	Neither intervention was superior	1 study, N=25; RR 0.35, 95% CI 0.12-1.01
10% povidone-iodine solution plus 50% salicylic acid plaster vs salicylic acid plaster alone	Physician assessment	Neither intervention was superior	1 study, N=30; RR 1.43, 95% CI 0.95-2.16

^a^N: number of participants.

^b^RR: risk ratio.

## Abbreviations

MC: molluscum contagiosum
MMR: measles, mumps, and rubella
